# 非小细胞肺癌PD-1/PD-L1的表达与*EGFR*突变相关性研究

**DOI:** 10.3779/j.issn.1009-3419.2021.102.31

**Published:** 2021-09-20

**Authors:** 玲 姜, 芷伊 林, 娜 李, 金芳 蒋, 层层 卢, 胜行 杜, 军 张, 圆圆 王, 军 陈, 平 巩

**Affiliations:** 1 832002 石河子，石河子大学医学院第一附属医院 First Affiliated Hospital, School of Medicine, Shihezi University, Shihezi 832002, China; 2 629000 遂宁，遂宁市中心医院 Suining Central Hospital, Suining 629000, China; 3 832002 石河子，石河子大学医学院 Clinical Medical School Shihezi University, Shihezi 832002, China; 4 300052 天津，天津医科大学总医院肺部肿瘤外科 Department of Lung Cancer Surgery, Tianjin Medical University General Hospital, Tianjin 300052, China

**Keywords:** 肺肿瘤, 程序性死亡受体1, 程序性死亡配体1, *EGFR*基因, 免疫组化, Lung neoplasms, Programmed death receptor 1, Programmed death receptor ligand 1, Epidermal growth factor receptor, Immunohistochemical

## Abstract

**背景与目的:**

肺癌的治疗模式以表皮生长因子受体酪氨酸激酶抑制剂（epidermal growth factor receptor-tyrosine kinase inhibitors, EGFR-TKIs）作为*EGFR*突变的非小细胞肺癌（non-small cell lung cancer, NSCLC）患者一线治疗；同时以程序性死亡受体1（programmed death receptor 1, PD-1）及其配体（programmed death receptor ligand 1, PD-L1）抑制剂为代表的免疫检查点抑制剂（immune checkpoint inhibitors, ICIs）的免疫治疗在肺癌治疗中疗效显著。本研究旨在探讨PD-1和PD-L1在NSCLC中的表达及其与临床病理特征、*EGFR*突变之间的关系。

**方法:**

采用免疫组化方法检测127例NSCLC PD-1和PD-L1蛋白表达，同时用定量聚合酶链反应（quantitative polymerase chain reaction, qPCR）检测*EGFR*基因突变，分析其与临床病理特征之间的关系，研究PD-1、PD-L1表达之间以及其与*EGFR*突变的关系。

**结果:**

NSCLC肿瘤细胞及肿瘤浸润免疫细胞PD-1阳性表达53.5%（68/127），肿瘤细胞PD-L1表达57.5%（73/127），PD-1和PD-L1的表达在低分化癌、临床分期Ⅰ期+Ⅱ期明显高于高中分化癌、Ⅲ期+Ⅳ期（均*P* < 0.05）；*EGFR*突变率为46.5%（59/127），*EGFR*突变的患者中女性、无吸烟史、腺癌、高中分化组分别高于男性、吸烟史、鳞癌、低分化组患者（均*P* < 0.05）；NSCLC患者PD-L1与PD-1蛋白表达存在一致性（kappa=0.107, 5, *P*=0.487），*EGFR*突变与PD-1、PD-L1表达存在负相关关系（Φ=-0.209，Φ=-0.221，均*P* < 0.05）；对NSCLC患者随访，在 < 65岁、腺癌、高中分化癌、PD-L1表达的患者中位总生存期分别高于≥65岁、鳞癌、低分化癌、PD-L1不表达患者（均*P* < 0.05）。PD-L1低表达患者中位生存期明显高于高表达患者（*P*=0.04）。

**结论:**

参照《非小细胞肺癌PD-L1免疫组织化学检测规范中国专家共识》检测非小细胞肺癌PD-L1表达，筛选出抗PD-1/PD-L1治疗的优势人群；同时检测出*EGFR*突变的患者，并且*EGFR*突变与PD-1、PD-L1表达存在负相关关系，依据PD-L1表达和*EGFR*突变状态，可能使NSCLC患者在的个体化治疗中获益，同时65岁以下、腺癌、高中分化、PD-L1低表达的患者有相对好的预后，为NSCLC预后评估提供参考。

## Abstract (en)

**Background and objective:**

The treatment mode of lung cancer is epidermal growth factor receptor-tyrosine kinase inhibitors (EGFR-TKIs) as a first-line treatment for patients with *EGFR* mutant in non-small cell lung cancer (NSCLC). At the same time programmed death receptor 1 (PD-1) and its programmed death receptor ligand 1 (PD-L1) inhibitors therapy as the representative immune checkpoint inhibitors (ICIs) has a significant effect in the treatment of lung cancer. The aim of this study was to investigate the correlation between the expression of PD-1 and PD-L1 in NSCLC and clinicopathologic feature, *EGFR* gene mutation.

**Methods:**

The protein expression of PD-1 and PD-L1 was detected by immunohistochemistry from 127 patients with NSCLC and *EGFR* gene mutation was detected by quantitative polymerase chain reaction (qPCR) to analyze its relation with clinicopathologic feature. Also, the correlation between protein expression of PD-1 and PD-L1 and *EGFR* mutation.

**Results:**

The PD-1 positive expression in NSCLC tumor cells and tumor infiltrating immune cells is 53.5% (68/127), PD-L1 is 57.5% (73/127). The PD-1 and PD-L1 expression significantly higher in well-differentiated and moderately-differentiated carcinoma than poorly differentiated carcinoma, Ⅰ+Ⅱ than Ⅲ+Ⅳ in clinical staging (*P* < 0.05). The *EGFR* mutation rate was 46.5% (59/127), correlate with female, without smoking history, adenocarcinoma and well-differentiated and moderately-differentiated patients respectively higher than male, smoking history, squamous carcinoma and poorly differentiated patients (*P* < 0.05). The protein expression of PD-L1 and PD-1 had the consistency in NSCLC patients (kappa=0.107, 5, *P*=0.487). There was a negative correlation between the *EGFR* mutation and PD-1 and PD-L1 expression (Φ=-0.209, Φ=-0.221, *P* < 0.05). Follow-up of NSCLC patients, the median total survival in under the age of 65, adenocarcinoma, well-differentiated and moderately-differentiated, with PD-L1 expression patients respectively higher than over the age of 65, squamous carcinoma, poorly differentiated, without PD-L1 expression patients (*P* < 0.05). The median survival of hypo expression patients of PD-L1 significantly higher than hyper expression patient (*P*=0.04).

**Conclusion:**

According to the Chinese Expert Consensus on Standards of PD-L1 immunohistochemistry testing for NSCLC, we tested the PD-L1 expression in NSCLC and then the dominant population of anti-PD-1/PD-L1 treatment was screened out. Patients with *EGFR* mutation were also detected and *EGFR* mutation was negatively correlated with the expression of PD-1 and PD-L1 as well. On the basis of PD-L1 expression and *EGFR* mutation status, it may benefit NSCLC patients from individualized treatment. Meanwhile, patients who were under the age of 65, adenocarcinoma, well-differentiated and moderately-differentiated, hypo expression of PD-L1 have a relatively good prognosis, to provide reference for the prognosis evaluation of NSCLC.

肺癌是严重威胁人类健康的恶性肿瘤，我国男性及女性肿瘤相关死亡率第1位和第2位^[1]^，其中非小细胞肺癌（non-small cell lung cancer, NSCLC）在肺癌中约占80%^[2]^，多数患者在确诊时已经晚期，无法手术，且系统化疗和放疗的作用有限。以吉非替尼、厄洛替尼、奥希替尼等为代表的表皮生长因子受体酪氨酸激酶抑制剂（epidermal growth factor receptor tyrosine kinase inhibitors, EGFR-TKIs）成为存在*EGFR*突变NSCLC患者的标准一线治疗方案^[3]^，其客观缓解率可达到70%左右甚至更高，但通常1年左右就会产生耐药^[4]^。以程序性死亡受体1（programmed death receptor 1, PD-1）及其配体（programmed death receptor ligand 1, PD-L1）抑制剂为代表的免疫检查点抑制剂（immune checkpoint inhibitors, ICIs）在肺癌治疗中取得突破性进展，尤其是PD-L1高表达的患者可以从中获益更多^[5]^，但相关研究却显示免疫检查点抑制剂对*EGFR*突变患者的治疗效果并不理想^[6-9]^。本研究依据《非小细胞肺癌PD-L1免疫组织化学检测规范中国专家共识》^[10]^对NSCLC进行免疫组化PD-L1、PD-1检测，用定量聚合酶链反应（quantitative polymerase chain reaction, qPCR）方法检测*EGFR*基因状态，探讨PD-1、PD-L1在NSCLC组织中表达与临床特征、*EGFR*基因突变之间的关系，希望在肺癌的个体化治疗及预后评估方面提供更多的参考。

## 1 材料与方法

### 1.1 材料

#### 1.1.1 病例收集及临床资料

收集石河子大学医学院第一附属医院病理科2016年4月-2019年8月确诊NSCLC患者127例，男性67例，平均年龄（69.62±9.61）岁。女性60例，平均年龄（69.1±9.87）岁。NSCLC患者临床信息包括性别、年龄、吸烟史、标本类型、组织学类型、分化程度，淋巴结转移及临床分期（参照第8版国际肺癌肿瘤淋巴结转移分期），排除既往行新辅助化疗或有过恶性肿瘤病史的患者，同时征求患者同意，并签署知情同意书。所研究组织标本经10%中性福尔马林固定6 h-24 h，常规取材、处理标本制成石蜡组织。患者一般资料见表 1。

**表 1:** 127例NSCLC临床信息 Clinicopathological features of 127 NSCLC patients

Category		*n* (%)
Gender	Male	67 (52.8)
	Female	60 (47.2)
Age (yr)	< 65	57 (44.9)
	≥65	70 (55.1)
Smoking status	Yes	57 (44.9)
	No	70 (55.1)
Specimen type	Percutancous lung biopsy	28 (22.0)
	Bronchoscopy	11 (8.7)
	Operation	73 (57.5)
	Metastasizeelsewhere	15 (11.8)
Pathological type	Adenocarcinoma	99 (78.0)
	Squamous cell carcinoma	19 (15.0)
	Other types	9 (7.0)
Tumor differentiation	High+middle	83 (65.4)
	Low	44 (34.6)
Lymphatic metastasis	Yes	88 (69.3)
	No	39 (30.7)
Clinical stage	Ⅰ+Ⅱ	95 (74.8)
	Ⅲ+Ⅳ	32 (25.2)
NSCLC: non-small cell lung cancer.		

#### 1.1.2 实验试剂及仪器

PD-1（鼠抗人单克隆抗体MRQ-22，即用型，北京中杉公司），二抗试剂（Envision试剂盒，DAKO，K5007）；PD-L1检测试剂盒（DAKO平台的PD-L1 IHC 22C3 PharmDX）包括：细胞株（包含PD-L1阳性、阴性），20×缓冲液、50×修复液，PD-L1即用型抗体，二抗试剂，DAB显色系统，每例检测样本包含两张切片（22C3Ab, 22C3NCR）。免疫组化修复仪（DAKO PTlink），自动免疫组化染色仪（DAKO Link 48），数字病理切片扫描仪（KF-PRO-005）。

qPCR试剂及仪器：福尔马林固定石蜡包埋组织DNA提取试剂盒（QIAGEN）和人*EGFR*基因外显子18-21突变检测试剂盒（北京雅康博生物医药科技股份有限公司）。核酸测定仪（NanoDrop 2000，美国Thermo公司），荧光定量PCR仪（7500Fast美国Life technologies公司）。

### 1.2 方法

#### 1.2.1 免疫组化PD-1检测

石蜡组织4 μm连续切片，石蜡切片常规脱蜡至水，Tris-EDTA高温高压修复3 min，3%H_2_O_2_溶液封闭10 min，加入PD-1抗体100 μL，4 ℃冰箱过夜，PBS浸洗5 min，加100 μL HRP标记的二抗37 ℃温箱30 min，PBS浸洗5 min，DAB显色，苏木素复染。PD-L1检测：常规切片脱蜡至水，修复仪修复40 min，缓冲液浸洗，玻片放入自动免疫组化染色机中进行染色，机器运行正常。

结果判定：用数字病理切片扫描仪扫描所有免疫组化切片，请两名经验丰富的病理医师判读扫描的切片。PD-1在肿瘤细胞及肿瘤浸润的免疫细胞胞浆染色，采用CPS评分[任意强度胞浆染色的肿瘤细胞和免疫细胞相对于肿瘤细胞（至少100个）的比例分数≥1即为阳性， < 1为阴性]，PD-L1定位于肿瘤细胞膜，阳性对照细胞株胞膜呈棕色，阴性对照细胞株无染色，22C3NCR（空白对照）无染色。按照DAKO PD-L1 IHC 22C3判读标准^[10]^，PD-L1以TPS（任何强度的部分或完全膜染色的肿瘤细胞占标本中所有肿瘤细胞的百分比）作为表达结果，病理医师对所有PD-L1染色进行肿瘤细胞阳性比例分数（tumor proportion score, TPS）评分，TPS < 1%为阴性，TPS≥1%为阳性表达，其中TPS 1%-49%为低表达，TPS≥50%为高表达。

#### 1.2.2 石蜡组织DNA的提取与质控

通过HE切片对NSCLC组织进行评估，按照肿瘤细胞数量及百分比，活检标本5 μm 4张-8张，穿刺标本5 μm 10张-15张，用石蜡包埋组织DNA提取试剂盒，操作步骤按照QIAGEN公司说明书提取DNA，使用核酸测定仪测定每个样本DNA纯度和浓度，并记录，样本DNA的A_260_/A_280_比值介于1.8-2.1为有效。

#### 1.2.3 *EGFR*基因外显子18-21突变检测

依据各样本测得浓度，将所有样本稀释到0.5 ng/µL，按照*EGFR*基因检测试剂说明书操作，用突变扩增系统（amplification refractory mutation system, ARMS）法检测*EGFR*外显子18-21共29个突变位点。每次实验均设阴性、阳性对照及空白对照，根据说明书PCR反应扩增曲线及CT值进行分析。

### 1.3 随访

课题组从2017年3月1日陆续对纳入我们研究的NSCLC患者进行随访，采用门诊随访和电话随访相结合的方式，获取患者生存资料，截止到2020年10月25日，有90例患者随访信息完整。

### 1.4 统计学分析

应用SPSS 20.0软件对数据进行统计分析。NSCLC组织中PD-L1和PD-1表达、*EGFR*基因突变状态与临床病理特征等分类资料指标采用频数和百分比进行描述，组间的差异比较采用卡方（或秩和）检验，组间的相关性采用*McNemar*和列联系数进行相关性分析，用*Kaplan-Meier*曲线分析各因子与NSCLC患者生存情况的关系，所有检验均为双侧检验，以*P* < 0.05为差异有统计学意义。

## 2 结果

### 2.1 PD-1、PD-L1表达与临床病理特征的关系

PD-1的蛋白表达在肿瘤细胞及肿瘤浸润的免疫细胞胞浆（见图 1A-图 1D），127例NSCLC患者中，PD-1表达阳性率为53.5%（68/127），肿瘤细胞和免疫细胞都表达的阳性率为46.5%（59/127），肿瘤细胞表达、免疫细胞不表达为3.9%（5/127），肿瘤细胞不表达、免疫细胞表达为3.1%（4/127），肿瘤细胞和免疫细胞都不表达为46.5%（59/127）。PD-L1在肿瘤细胞中部分或完全膜染色（图 1E-图 1G），阳性表达率为57.5%（73/127），其中高表达占11%（14/127），低表达占46.5%（59/127）；本研究NSCLC患者PD-1、PD-L1的表达都与肿瘤分化程度、临床分期有统计学差异，与其他临床指标均无统计学差异。PD-1在低分化癌组织中的表达率为65.9%（29/44），高于高、中分化癌中的表达率47.0%（39/83）（*P*=0.042），PD-1在临床分期Ⅰ期和Ⅱ期的阳性表达率为60%（57/95），高于Ⅲ期和Ⅳ期的阳性表达率34.4%（11/32）（*P*=0.012）；PD-L1在低分化癌组织中的表达率为70.5%（31/44），高于高、中分化癌中的表达率50.6%（42/83）（*P*=0.031）；PD-L1在临床分期Ⅰ期和Ⅱ期的阳性表达率为63.2%（60/95），高于Ⅲ期+Ⅳ期的阳性表达率40.6%（13/32）（*P*=0. 026）（表 2）。

**图 1:**
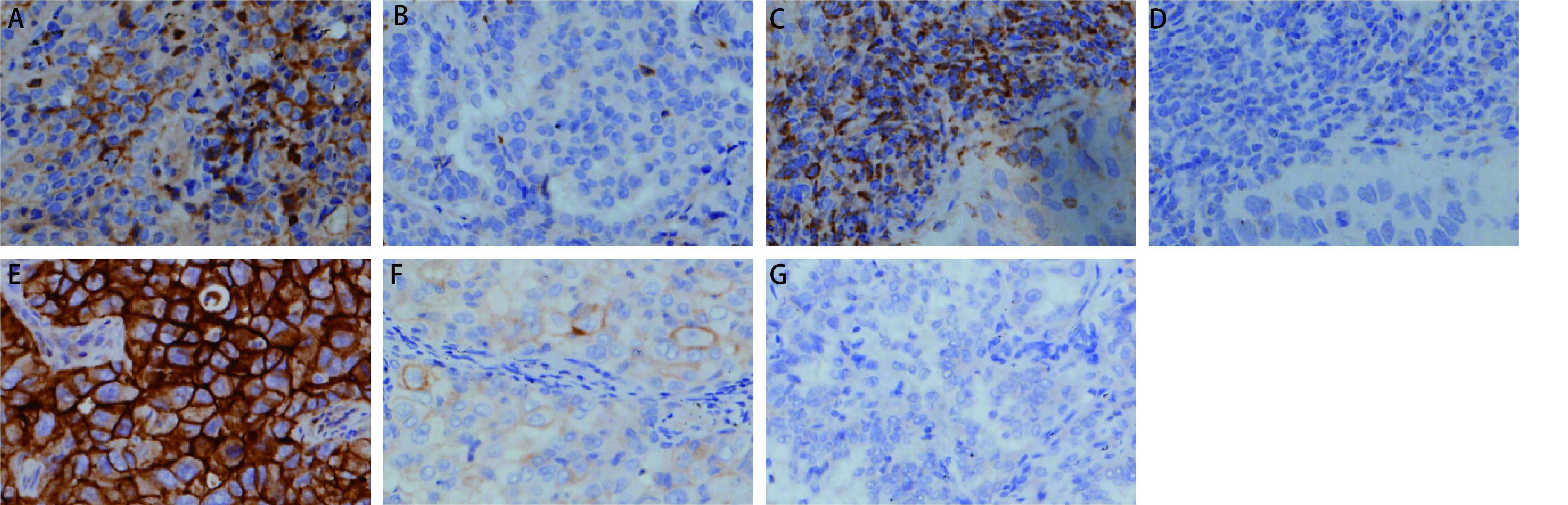
PD-1和PD-L1在NSCLC中的表达。A：PD-1在肿瘤细胞中的表达（×200）；B：PD-1在肿瘤细胞中不表达（×200）；C：PD-1在免疫细胞中表达（×200）；D：PD-1在免疫细胞中不表达（×200）；E：PD-L1在肿瘤细胞中高表达（×200）；F：PD-L1在肿瘤细胞中低表达（×200）；G：PD-L1在肿瘤细胞中不表达（×200）。 Expression of PD-1 and PD-L1 in NSCLC. A: PD-1 expression in tumor cells (×200); B: PD-1 not expressed in tumor cells (×200); C: PD-1 expressed in immune cells (×200); D: PD-1 not expressed in immune cells (×200); E: PD-L1 was high expressed in tumor cells (×200); F: PD-L1 was low expressed in tumor cells (×200); G: PD-L1 not expressed in tumor cells (×200). PD-1: programmed death receptor 1; PD-L1: programmed death receptor ligand 1.

**表 2:** PD-1和PD-L1在NSCLC中的表达与临床特征的关系（*n*=127）[*n*(%)] Correlation of expression of PD-1 and PD-L1 with clinical features in NSCLC (*n*=127) [*n*(%)]

Features	*n*	PD-1		*χ* ^2^	*P*	PD-L1		*χ* ^2^	*P*
		Positive	Negative			Positive	Negative		
Gender				0.574	0.449			0.031	0.860
Male	67	38 (56.7)	29 (43.3)			39 (58.2)	28 (41.8)		
Female	60	30 (50.0)	30 (50.0)			34 (56.7)	26 (43.3)		
Age (yr)				1.307	0.253			0.286	0.593
< 65	57	39 (68.4)	18 (31.6)			40 (70.2)	17 (29.8)		
≥65	70	41 (58.6)	29 (41.4)			46 (65.7)	24 (34.3)		
Smoking status				0.787	0.375			0.833	0.361
Yes	57	33 (57.9)	24 (42.1)			33 (57.9)	24 (42.1)		
No	70	35 (50.0)	35 (50.0)			40 (57.1)	30 (42.9)		
Pathological type				0.346	0.841			3.156	0.206
Adenocarcinoma	99	54(54.5)	45 (45.5)			61 (61.6)	38 (38.4)		
Squamous cell carcinoma	19	9 (47.4)	10 (52.6)			8 (42.1)	11 (57.9)		
Other types	9	5 (55.6)	4 (44.4)			4 (44.4)	5 (55.6)		
Tumor differentiation				4.139	0.042			4.637	0.031
High+middle	83	39 (47.0)	44 (53.0)			42 (50.6)	41 (49.4)		
Low	44	29 (65.9)	15 (34.1)			31 (70.5)	13 (29.5)		
Lymphatic metastasis				2.24	0.134			0.028	0.866
Yes	88	51 (58.0)	37 (42.0)			60 (68.2)	28 (31.8)		
No	39	17 (43.6)	22 (56.4)			26 (66.7)	13 (33.3)		
Clinical stage				6.319	0.012			4.97	0.026
Ⅰ+Ⅱ	95	57 (60.0)	38 (40.0)			60 (63.2)	35 (36.8)		
Ⅲ+Ⅳ	32	11 (34.4)	21 (65.6)			13 (40.6)	19 (59.4)		

### 2.2 *EGFR*突变状态与患者临床特征的关系

127例NSCLC患者中*EGFR*基因突变59例，突变率为46.5%（59/127），突变位点涉及到外显子18、19、20及21，其中点突变54例，19-Del突变27例（45.7%）和L-858R 23例（39%），18 G719X和20 Ins突变各1例；双重突变5例，19-Del与20 T790M、21 L-858R突变分别为2例、1例，20 T790M与21 L-858R双突变2例。127例NSCLC患者中，女性患者*EGFR*基因突变率58.3%（35/60）高于男性35.8%（24/43）（*P*=0.011）、无吸烟史55.7%（39/70）高于吸烟史35.1%（20/57）（*P*=0.020）、腺癌54.5%（54/99）高于鳞癌21.1%（4/19）和其他类型癌11.1%（1/9）（*P*=0.002）、高、中分化癌患者53.0%（44/83）高于低分化癌患者34.1%（15/44）（*P*=0.042）（表 3）。

**表 3:** NSCLC患者*EGFR*基因突变与临床特征的关系（*n*=127）[*n*(%)] Relationship between *EGFR* gene mutation and clinical characteristics in NSCLC patients (*n*=127) [*n*(%)]

Features	*n*	*EGFR* wild type	*EGFR* mutant	*χ* ^2^	*P*
Gender				6.449	0.011
Male	67	43 (64.2)	24 (35.8)		
Female	60	25 (41.7)	35 (58.3)		
Age (yr)				0.368	0.544
< 65	57	23 (40.4)	34 (59.6)		
≥65	70	32 (45.7)	38 (54.3)		
Smoking status				5.374	0.020
Yes	57	37 (64.9)	20 (35.1)		
No	70	31 (44.3)	39 (55.7)		
Pathological type				12.054	0.002
Adenocarcinoma	99	45 (45.5)	54 (54.5)		
Squamous cell carcinoma	19	15 (78.9)	4 (21.1)		
Other types	9	8 (88.9)	1 (11.1)		
Tumor differentiation				4.139	0.042
High+middle	83	39 (47.0)	44 (53.0)		
Low	44	29 (65.9)	15 (34.1)		
Lymphatic metastasis				0.002	0.960
Yes	88	47 (53.4)	41 (46.6)		
No	39	21 (53.8)	18 (46.2)		
TNM stage				0.126	0.723
Ⅰ+Ⅱ	95	50 (52.6)	45 (47.4)		
Ⅲ+Ⅳ	32	18 (56.2)	14 (43.8)		
EGFR: epidermal growth factor receptor; TNM: tumor-node-metastasis.					

### 2.3 PD-1、PD-L1蛋白表达之间及其与*EGFR*突变的相关性

采用*McNemar*分析PD-1与PD-L1相关性发现，PD-1与PD-L1都表达54例（74.0%），40例（74.1%）都不表达（kappa=0.107, 5, *P*=0.487）。进一步采用列联系数相关性分析*EGFR*突变与PD-1和PD-L1表达的关系，发现有59例*EGFR*突变的患者中PD-1，表达率为42.4%，不表达率为57.6%，说明*EGFR*突变与PD-1表达存在负相关关系（Φ=-0.209, *P*=0.019）；同样*EGFR*突变与PD-L1表达存在负相关关系（Φ=-0.221, *P*=0.013）（表 4）。

**表 4:** *EGFR*基因突变与PD-1、PD-L1蛋白表达的相关性（*n*=127）[*n*(%)] Correlation between EGFR gene mutation and PD-1 and PD-L1 protein expression (*n*=127) [*n*(%)]

Category	*n*	PD-1		*χ* ^2^	*P*	Φ	PD-L1		*χ* ^2^	*P*	Φ
		Negative	Positive				Negative	Positive			
*EGFR* wild type	68	25 (36.8)	43 (63.2)	0.428	0.019	-0.209	22 (32.4)	46 (67.6)	0.404	0.013	-0.221
*EGFR* mutant	59	34 (57.6)	25 (42.4)				32 (54.2)	27 (45.8)			

### 2.4 NSCLC患者的临床病理特征、PD-1、PD-L1的蛋白表达、*EFGR*突变与预后的相关因素分析

使用*Kaplan-Meier*生存曲线分析显示，NSCLC患者的总生存时间（overall survival, OS）与年龄在65岁以下患者中位OS（20个月）明显高于65岁以上患者（17个月）（*P*=0.008）；高、中分化癌患者中位OS（20个月）明显高于低分化癌患者（15个月）（*P*=0.033）；PD-L1表达的患者中位OS（20个月）明显高于PD-L1不表达患者（12个月）（*P* < 0.001），在PD-L1阳性的患者中低表达患者中位OS（22个月）明显高于高表达患者（15个月）（*P* < 0.001），腺癌中位OS（20个月）明显高于鳞癌（15个月）（*P*=0.042）（图 2，表 5）。

**图 2:**
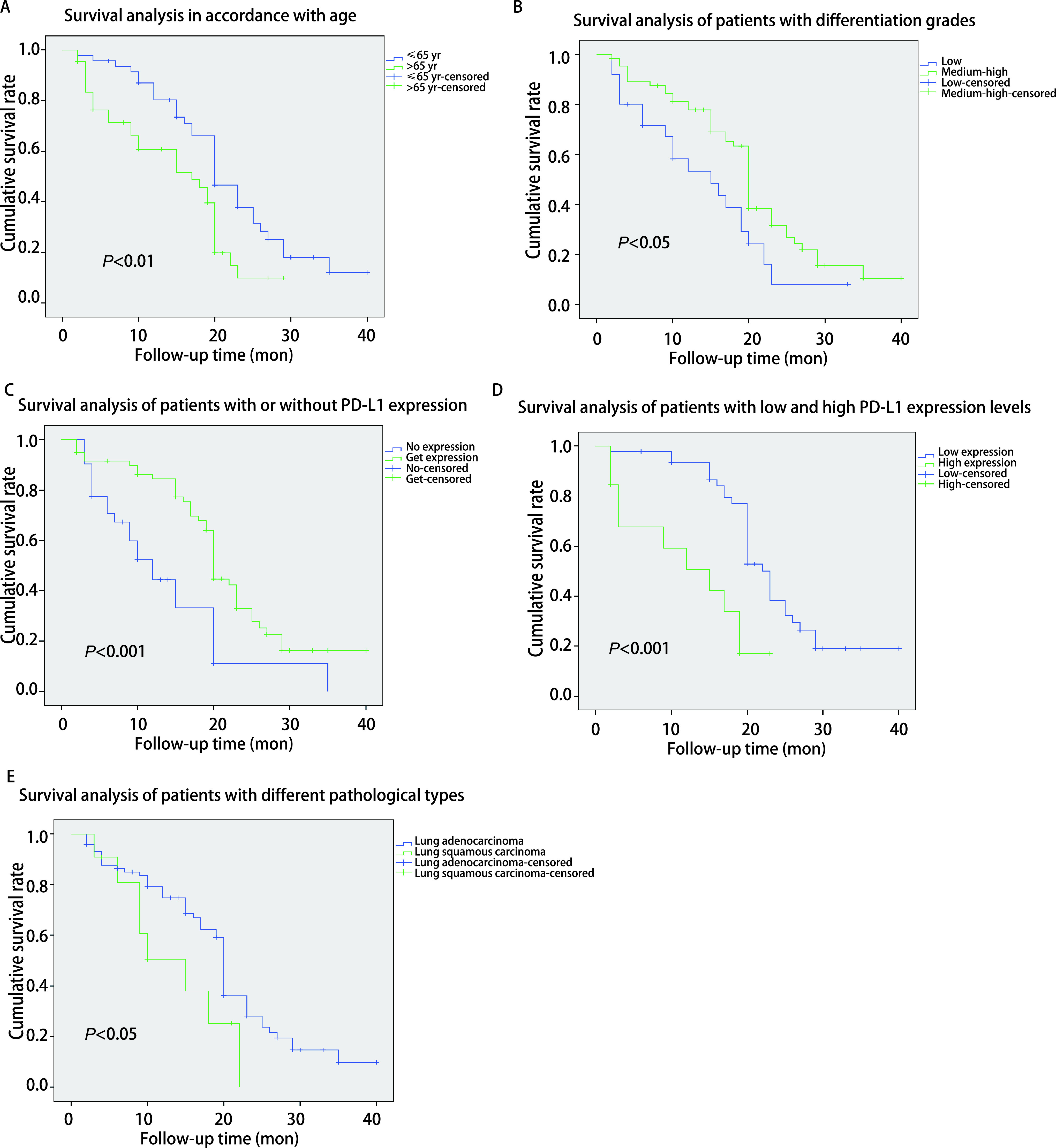
NSCLC癌患者中年龄、分化程度、PD-L1表达、肺腺癌和鳞癌与中位生存期之间关系。A：年龄65岁以下中位OS（20个月）明显高于65岁以上（17个月）；B：高、中分化癌患者中位OS（20个月）明显高于低分化癌患者（15个月）；C：PD-L1表达的患者中位OS（20个月）明显高于不表达的患者（12个月）；D：PD-L1低表达患者中位OS（22个月）明显高于高表达患者（15个月）；E：腺癌中位OS（20个月）明显高于鳞癌（15个月）。 The relationship between age, differentiation, PD-L1 expression, lung adenocarcinoma and squamous cell carcinoma and median survival in NSCLC patients. A: The median OS of people under 65 years old (20 mon) was significantly higher than that of people over 65 years old (17 mon); B: The median OS of patients with highly and moderately differentiated carcinoma (20 mon) was significantly higher than that of patients with poorly differentiated carcinoma (15 mon); C: The median OS of patients with PD-L1 expression (20 mon) was significantly higher than that of patients without PD-L1 expression (12 mon); D: The median OS of patients with low PD-L1 expression (22 mon) was significantly higher than that of patients with high PD-L1 expression (15 mon); E: The median OS of adenocarcinoma (20 mon) was significantly higher than squamous cell carcinoma (15 mon). OS: overall survival.

**表 5:** NSCLC生存期相关因素的单因素分析(*n*=90) Univariate analysis of factors related to survival of NSCLC (*n*=90)

Category	*n* (%)	Median OS (mon)	*χ* ^2^	*P*
Gender			2.230	0.135
Male	48 (53.3)	19		
Female	42 (46.7)	20		
Age (yr)			8.056	0.005
< 65	47 (52.2)	20		
≥65	43 (47.8)	17		
Tumor differentiation			5.942	0.015
Low	25 (27.8)	15		
High+middle	65 (72.2)	20		
*EGFR* mutations			0.589	0.443
No	53 (58.9)	17		
Yes	37 (41.1)	20		
PD-1 expression			0.040	0.842
No	37 (41.1)	20		
Yes	53 (58.9)	20		
PD-L1 expression			12.605	< 0.001
No	31 (34.4)	12		
Yes	59 (65.6)	20		
PD-L1 expression			14.844	< 0.001
Low expression	46 (78.0)	22		
High expression	13 (22.0)	15		
Pathological type			4.136	0.042
Adenocarcinoma	11 (12.2)	15		
Squamous cell carcinoma	74 (82.2)	20		

## 3 讨论

肺癌是全球癌症相关死亡的主要原因，肺癌的治疗已经全面进入精准医学时代，在NSCLC治疗中，EGFR-TKI已成为*EGFR*敏感突变晚期NSCLC的一线治疗。尽管如此，靶向治疗出现获得性耐药不可避免，并最终导致治疗失败。随着对肿瘤免疫逃逸机制的认识不断深入，对PD-1/PD-L1通路的免疫靶向药物在NSCLC治疗中已经表现出令人惊喜的效果。

PD-1是免疫球蛋白B7-CD28家族成员之一，主要在肿瘤浸润性淋巴细胞B淋巴细胞、自然杀伤（Natural kiiller, NK）T细胞、树突状细胞、单核-巨噬细胞表达^[11]^，PD-L1（B7-H1）及PD-L2（B7-DC）是PD-1的主要配体，其中PD-L2主要在抗原提呈细胞上表达，PD-L1在许多类型的细胞表达，包括肿瘤细胞，免疫细胞、上皮细胞和内皮细胞。

PD-L1与PD-1相结合，传递免疫抑制信号，抑制T细胞的活化与增殖，参与肿瘤免疫逃逸^[12]^。目前经美国食品药品监督管理局（Food and Drug Administration, FDA）批准可作为晚期NSCLC二线治疗的免疫检查点抑制剂主要有PD-1抗体Nivolumab、Pembrolizumab和PD-L1抗体Atezolizumab，其中Pembrolizumab 2016年获批成为NSCLC的一线治疗用药，用药效果与PD-L1的表达水平相关。研究Keynote-024表明驱动基因阴性的PD-L1≥50% NSCLC一线使用帕博利珠单抗单药治疗显著优于化疗，可使患者获益显著，中位无进展生存时间和总生存期与化疗组相应延长，且不良反应较化疗少^[13]^，而在PD-L1表达在1%-49%的患者则获益有限，随着PD-L1表达的增高，患者使用帕博利珠单抗治疗的OS逐步延长^[14]^。

研究^[13]^显示NSCLC中PD-1为29.2%-75.0%，PD-L1的阳性表达率为39.9%-53.1%。本研究中PD-1的表达采用CPS评分，为53.5%（68/127），肿瘤细胞和免疫细胞都表达为46.5%（59/127）；依照《非小细胞肺癌PD-L1免疫组织化学检测规范中国专家共识》^[10]^对样本进行PD-L1检测，包括推荐试剂、仪器、检测结果判读等都参照专家共识，PD-L1表达率为57.5%（73/127），其中PD-L1高表达占11%（14/127），低表达占46.5%（59/127），高锋等^[14]^PD-L1在NSCLC中阳性表达率为61.67%，在不同研究中PD-L1在NSCLC中的表达存在的异质性，在手术标本与活检标本间存在一定异质性^[15]^，国内相关研究同样显示类似结果^[16]^，本研究样本也存在手术标本57.5%，穿刺与支气管镜标本30.7%，肺癌转移标本11.8%，了解标本间PD-L1表达的异质性对临床检测有着重要的指导作用。

PD-1与PD-L1表达的相关性分析（kappa=0.107, 5, *P*=0.487）说明PD-1与PD-L1表达存在一致性，同时PD-1与PD-L1表达一致性还表现在临床病理特征的关系中，PD-1与PD-L1的表达与性别、年龄、吸烟史、组织类型、淋巴结转移均无统计学意义；在分化程度和临床分期中，PD-1与PD-L1在低分化癌中的表达65.9%（29/44）、70.5%（31/44）高于高、中分化癌47.0%（39/83）、50.6%（42/83），在临床分期Ⅰ期和Ⅱ期的阳性表达率60%（57/95）、63.2%（60/95）高于Ⅲ期和Ⅳ期的阳性表达率34.4%（11/32）、40.6%（13/32）（均*P* < 0.05）；高锋等^[14]^研究PD-L1在NSCLC中的表达与淋巴结转移、肿瘤细胞分化程度、TNM分期及生存期有关（P < 0.05），其中该作者PD-L1在低分化癌中的表达83.3%（15/18）高于高、中分化癌52.4%（22/42），但在临床分期中Ⅲ期（75.0%, 9/12）和Ⅱ期（70.9%, 22/31）表达高于Ⅰ期（35.3%, 6/17），PD-L1在临床分期中的表达差异，可能存在样本量少、各组样本不均衡，导致统计结果不一致。

*EGFR*基因位于第7号染色体，共有28个外显子。突变主要发生在外显子18-21，其中外显子19和21突变更为重要^[17]^。本研究发现*EGFR*在NSCLC中的突变率为46.5%（59/127），其中点突变54例，大部分为19-Del（27例，45.7%）和L-858R（23例，39%）。127例NSCLC患者中，女性、无吸烟史、腺癌、高中分化患者的*EGFR*突变率分别高于男性、有吸烟史、鳞癌、低分化患者，差异有统计学意义（P < 0.05）；丁光贵等^[18]^研究表明*EGFR*基因突变患者中女性和不吸烟患者的比例显著升高；本研究中*EGFR*突变患者PD-L1和PD-1的阳性率均低于野生型，*EGFR*突变与PD-1和PD-L1表达呈负相关（Φ＝-0.209，Φ＝-0.221，均*P* < 0.05）。Huynh等^[19]^研究发现*EGFR*突变与PD-L1表达呈负相关，Inoue等^[20]^研究同样发现*EGFR*突变可以下调PD-L1表达；而嵇晓辉等^[21]^、Ameratunga等^[22]^研究表明NSCLC组织中*EGFR*突变与PD-L1、PD-1表达与无相关性。但相关研究却显示免疫检查点抑制剂对*EGFR*突变患者的治疗效果并不理想^[6-9]^，推断前者的结论有一定的可靠性，但关于*EGFR*突变是如何影响PD-L1表达水平，这方面的机制需要进一步探索，为*EGFR*突变患者找到提高PD-1/PD-L1抑制剂治疗疗效的突破点。

对127例NSCLC患者进行随访，90例患者信息完整，年龄65岁以下、高、中分化癌患者中位生存时间明显高于65岁以上、低分化癌患者；同时PD-L1表达的患者中位生存时间明显高于不表达患者，PD-L1低表达患者中位生存期明显高于高表达患者。本研究中腺癌在NSCLC中所占比例高，其他类型比较少，对腺癌和鳞癌患者生存期进行分析，腺癌OS明显高于鳞癌。本研究由于样本量相对少，失访的患者可能对统计数据造成一些影响，但随访结果还是有一定的参考价值。

本研究依照《非小细胞肺癌PD-L1免疫组织化学检测规范中国专家共识》对127例NSCLC组织进行PD-L1检测、判读，筛选出PD-L1高表达和低表达的患者，能够使这些患者在抗PD-1/PD-L1免疫治疗中获益，从而提高生存率；PD-1、PD-L1的表达都与肿瘤分化程度、临床分期有关，*EGFR*突变与PD-1、PD-L1存在负相关的关系，临床医生依据*EGFR*基因状态和免疫检测点PD-L1表达情况选择合适的治疗方案。同时本研究中65岁以下、腺癌、高、中分化、PD-L1表达的患者有较好的预后，为NSCLC预后评估提供依据。
